# A comparative study of paranasal sinus and nasal cavity anatomic variations between the Polish and Turkish Cypriot Population with CBCT

**DOI:** 10.1186/s13005-022-00340-3

**Published:** 2022-11-26

**Authors:** Katarzyna Gruszka, Seçil Aksoy, Ingrid Różyło-Kalinowska, Melis Mısırlı Gülbeş, Paweł Kalinowski, Kaan Orhan

**Affiliations:** 1 Private Clinic, Frampol, Poland; 2 Faculty of Dentistry, Department of Dentomaxillofacial Radiology, Near East University, 99138 Mersin 10, Turkey; 3 Department of Dental and Maxillofacial Radiodiagnostics, Medical University of Lublin, 20-093 Lublin, Poland; 4 Department of Dentomaxillofacial Radiology, International Final University, 99138 Mersin 10, Turkey; 5 Department of Hygiene and Epidemiology, Medical University of Lublin, 20- 093 Lublin, Poland; 6 Faculty of Dentistry, Department of Dentomaxillofacial Radiology, Ankara University, 06100 Ankara, Turkey; 7 Medical Design Application and Research Center (MEDITAM), Ankara University, 06100 Ankara, Turkey

**Keywords:** Sinonasal anatomy, Variations, Climate, CBCT

## Abstract

**Background:**

Genetic and environmental factors especially climatic conditions are thought to influence the shape and size of the paranasal sinuses and anatomic variations may create both a diagnostic and therapeutic challenge. However, no study has been published about the climatic adaptation of the paranasal sinus region in different populations. This study aimed to compare the prevalence of anatomical variants in the paranasal sinus and nasal cavity using Cone-Beam Computed Tomography (CBCT) between Polish and Turkish Cypriot populations.

**Methods:**

The material consisted of volumes acquired utilizing Galileos (Sirona, Germany) as well as Newtom 3G (QR Verona, Newtom, Italy) CBCT units. There were examined 356 Polish and 359 Turkish Cypriot patients in whom paranasal sinuses were included in the field of view. Paranasal sinus anatomic variations were assessed in both populations.

**Results:**

In the Polish population, the most common anatomic variation was septum deviation followed by the Agger nasi cell and concha bullosa with a prevalence of 87.7%, 83.2%, and 54.8% respectively. For the Turkish Cypriot population, the most common anatomic variation was Agger nasi cell followed by concha bullosa and supraorbital ethmoid cells with a prevalence of 81.6%, 68%, and 57.8% respectively. Many anatomic variations were found to show substantial differences among both populations. Incidence rates of hyperpneumatization of the frontal sinus, septum pneumatization, supraorbital ethmoid cells, concha bullosa, uncinate bulla, and internal carotid artery protrusion into the sphenoid sinus were significantly higher in the Turkish Cypriot group, while the incidence of Haller cell, frontal sinus hypoplasia, maxillary sinus hypoplasia, ethmomaxillary sinus, sphenomaxillary plate, and septum deviation were significantly higher in Polish population.

**Conclusion:**

According to the Köppen-Geiger world climatic map, the climate is warmer and drier in Turkish Cypriote populations than in the Polish population. These climatic differences influence the paranasal sinus variations between the Turkish Cypriot and Polish populations that must be taken into account by rhinologic surgeons especially when performing frontal and sphenoid sinus surgery.

## Background

The formation and development of the paranasal sinuses begin with the excavation of the air-filled cavity to the bone in the early weeks of gestation and reach their final size until adolescence. During the developmental process of the paranasal sinuses, anatomic variations may occur owing to the intramural and extramural migrations of the ethmoid air cells and overpneumatization of the sinuses as well as bulging of the neurovascular structures to the sinuses [1].

Genetic and environmental factors are thought to influence the occurrence of anatomic variations. The paranasal sinus anatomic variations study in identical and non-identical twins finds out that environmental factors are much more significant than genetic factors [2]. Although some anatomic variations such as nasal septum deviation and supreme nasal concha may exist already in the third trimester of the human fetuses, most of the anatomic variations occur due to trauma and/or chronic infections [3].

Some anatomic variations such as Haller cell, Agger nasi cell, concha bullosa, and nasal septum deviation may induce the obstruction in the drainage pathways of the maxillary and frontal sinuses and predispose the chronic rhinosinusitis. However, the relationship between the anatomic variations and chronic sinusitis remains controversial in the literature [4–9]. Paranasal sinus anatomic variations, even if they do not associate with rhinosinusitis, should also be evaluated to prevent the fatal and major complications secondary to sinus surgery. Moreover, identifying anatomical and morphological relationships is crucial for the surgeon when approaching the skull base to avoid the risk of iatrogenic injuries in these thinnest and most vulnerable structures [10].

Many imaging modalities including plain radiographs [11], computed tomography (CT) [4, 6, 8, 12–14], and cone-beam computed tomography (CBCT) [15–17] are used for the evaluation of the detailed anatomy, pathology, and variations of the paranasal sinuses. Although CT is established as the gold standard for diagnostic imaging of the paranasal sinuses, CBCT has advantages in terms of lower radiation dose, isotropic voxels, lower metallic artifacts, and lower cost than CT [18].

Paranasal sinuses and associated neurovascular structures are very important for otorhinolaryngologists and neurosurgeons however dentists are relatively neglecting this important area. Many studies about the paranasal sinus anatomic variations have been published in specific populations [4, 6, 8, 16] but few studies have focused on the ethnic differences [13, 14]. Previous investigations exploring the climatic and geographic changes of the nasal region have largely concentrated on the nasal morphology, size, and shape of the nasal aperture and internal nasal valve. This study aimed to assess and compare the prevalence of paranasal sinus anatomic variations, and the effect of geography and climate on paranasal sinus anatomic variety, using CBCT in Turkish Cypriot and Polish populations.

## Methods

This retrospective study comprised 356 Polish and 359 Turkish Cypriot patients’ CBCT images which included the paranasal sinus region. Patients with evidence of bone disease, relevant drug consumption, skeletal asymmetries or trauma, congenital disorders, anamnesis of surgical procedures, and pathological disorders of the maxilla as well as syndromic patients were excluded from the study.

The study protocol was carried out according to the principles described in the Declaration of Helsinki, including all amendments and revisions. Only the investigators had access to the collected data. The institutional ethical review board of the faculty reviewed and approved informed consent forms and the study (IRB No: YDU/25–147). There was no preference for gender regarding sample choice; however, only high-quality scans were included. Low-quality images, such as those containing scattering or insufficient accuracy of bony borders, were also excluded.

### Imaging using CBCT

For the Polish population, all CBCT images were taken by Galileos (Sirona, Germany) unit at 85 kVp and 14 mA with 0.3 mm slice thickness using a large field of view (FOV) (15 cm in diameter) and by Newtom 3G (QR Verona, Newtom, Italy) at 120 kVp and 3–5 mA using a 12 or 9-inch FOV with 0.4 mm slice thickness for Turkish Cypriot population. While the Polish group consisted of 135 males and 221 females with an age range of 6 to 89 years (mean 38.4 years), the Turkish Cypriot group included 170 males and 189 females with an age range of 6 to 89 years (mean 40.2 years). Axial, sagittal, and coronal images were reconstructed and assessed for paranasal sinus variations. All CBCT images were evaluated by two dentomaxillofacial radiologists (SA and KG) for the presence of the paranasal sinus variations listed in Table 1. An additional evaluation was held by the other two observers (IRK and KO) in which no consensus was reached. The results were analyzed through the Statistica for Windows software. Chi^2^ test was used and the level of significance was *p*˂0.05.

**Table 1:** Definitions of the paranasal sinus anatomic variations

Variations of the paranasal sinus	Definition
Frontal sinus hyperpneumatization	significant extension to the orbital plate and squamous part of the frontal bone
Frontal sinus hypoplasia	Sinus is devoid of pneumatization more than 6 mm in anteroposterior direction with not much extension to the squamous part of the frontal bone
Interfrontal sinus septa cells	pneumatization of the frontal sinus septum
Agger nasi cells	the most anterior ethmoid cells extramural migration to the frontal process of the maxilla
Supraorbital ethmoid cells	anterior ethmoid cells extending to the roof of the orbit and located behind the posterior wall of the frontal sinus
Crista galli pneumatization	air cavity in crista galli most commonly coming from the frontal sinus
Inferior concha bullosa	air filled cavity in the inferior turbinate
Haller cell	posterior ethmoid cells extramural migration to the floor of the orbit and roof of the maxillary sinus near ostium
Concha bullosa	air filled cavity of the middle turbinate
Uncinate bulla	pneumatization of the uncinate process
Secondary middle turbinate	accessory middle concha
Maxillary sinus hypoplasia	when the maximum horizontal or vertical diameter is less than half of the maximum orbital diameter.
Superior concha bullosa	air filled cavity in the superior turbinate
Secondary superior concha	accessory superior concha
Ethmomaxillary sinus	enlarged posterior ethmoid cells located over the maxillary sinus and draining into the superior meatus
Sphenomaxillary plate	bony plate lying between the maxillary and sphenoid sinus, triangular in shape
Septum deviation	developmental or acquired deviation in nasal septum
Septum pneumatization	pneumatization of the nasal septum
Internal carotid artery (ICA) bulging	projection of the ICA into the sphenoid sinus
Onodi cell	posterior ethmoid cell located in the anterosuperior portion of the sphenoid sinus and closely related to the optic nerve
Anterior clinoid pneumatization	air filled cavity in the anterior clinoid process
Posterior clinoid pneumatization	air filled cavity of the posterior clinoid process
Pterygoid pneumatization	extensive pneumatization of the sphenoid sinus which passes over the line between the foramen rotundum and Vidian canal

## Results

**Fig. 1:**
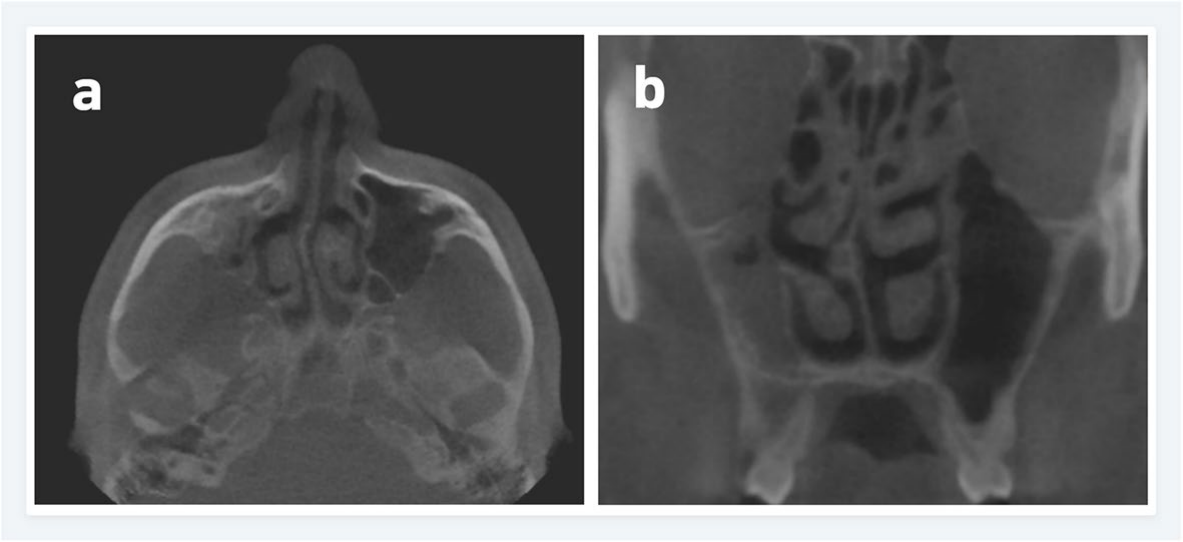
CBCT images of the nasal cavity and paranasal sinuses. Axial (**a**) and coronal (**b**) planes showing right side nasal septum deviation with septal spur (**b**) and maxillary sinus hypoplasia on the right side

**Fig. 2:**
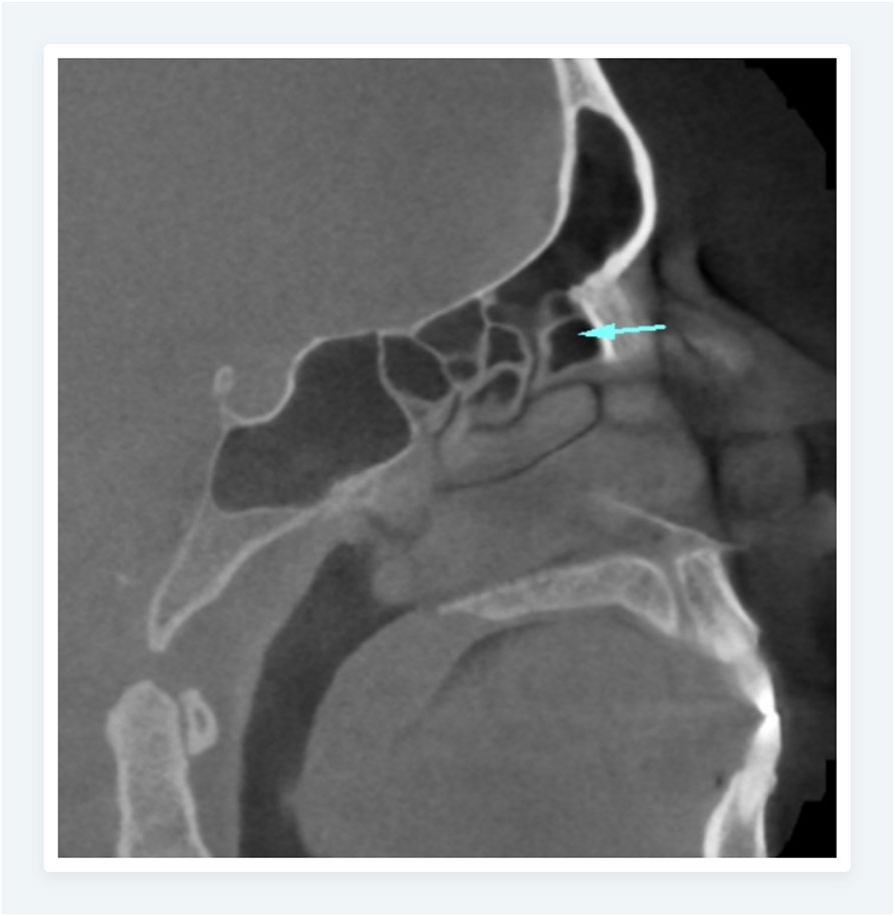
Sagittal CBCT plane showing agger nasi cell (arrow) and its relation with the frontal sinus ostium

**Fig. 3:**
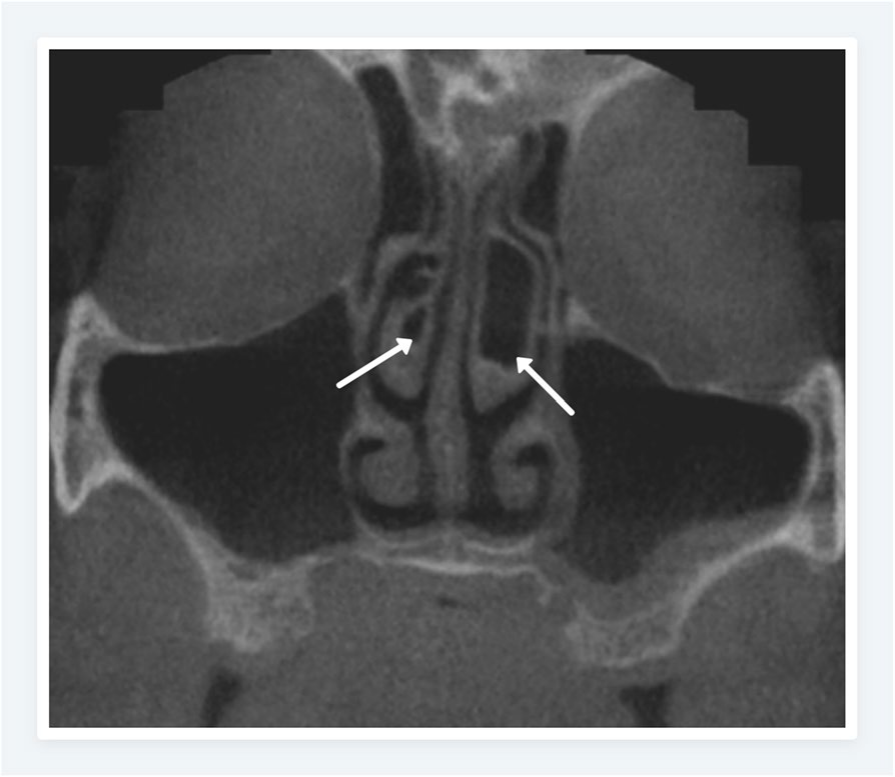
Coronal CBCT image showing the bulbous (right) and extensive (left) type concha bullosa with maxillary sinus mucosal thickening on the left side

**Fig. 4:**
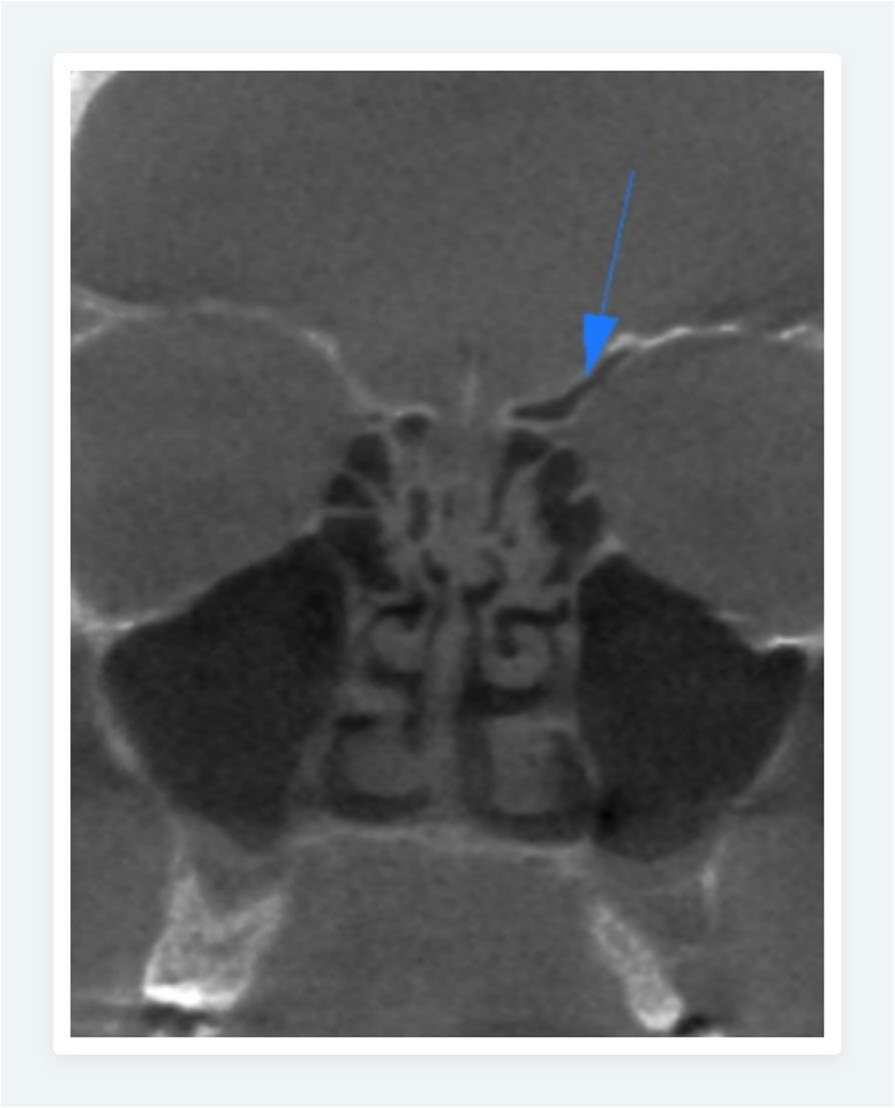
Coronal CBCT scan of the left supraorbital ethmoid cell

The prevalence of the anatomic variations in Polish and Turkish Cypriot Populations is shown in Table 2. In the Polish population, the most common anatomic variation was septum deviation (Fig. 1) followed by the Agger nasi cell (Fig. 2) and concha bullosa (Fig. 3) with a prevalence of 87.7%, 83.2%, and 54.8% respectively. For the Turkish Cypriot population, the most common anatomic variation was Agger nasi cell followed by concha bullosa and supraorbital ethmoid cells (Fig. 4) with a prevalence of 81.6%, 68%, and 57.8% respectively (Table 2).

**Table 2:** Comparison of the anatomic variations in Polish and Turkish Cypriot populations

Variations	Polish Population			Turkish Cypriot Population			*P* value
Frontal sinus hyperpneumatization	**4.5%** ^*^			**22.9%** ^*^			**˂ 0,05**
Frontal sinus hypoplasia	**16.7%** ^*^			**7.6%** ^*^			**˂ 0,05**
Interfrontal sinus septa cell	44.1%			41.8%			˃0,05
Crista galli pneumatization	4.2%			4%			˃0,05
Septum pneumatization	**44.1%** ^*^			**54%** ^*^			**˂ 0,05**
	**Right**	**Left**	**Both**	**Right**	**Left**	**Both**	
Agger nasi cell	16%	12.1%	55.1%	14.5%	13.1%	54.1%	˃0,05
Supraorbital ethmoid cell	**3.1%** ^*^	**3.9%** ^*^	**25.4%** ^*^	**7.9%** ^*^	12.2%^*^	**37.7%** ^*^	**˂ 0,05**
Inferior concha bullosa	0.8%	0.8%	1.4%	0.8%	1.4%	-	˃0,05
Concha bullosa	11%	12.1%	**31.7%** ^*^	14.5%	12.8%	40.7%^*^	˂ 0,05
Superior concha bullosa	9.6%	8.7%	19.7%	8.8%	13%	18.1%	˃0,05
Haller cell	8.4%	8.4%	**23.9%** ^*^	9.2%	11.2%	**15.6%** ^*^	**˂ 0,05**
Uncinate bulla	**3.7%** ^*^	**2%** ^*^	20.5%	**11.7%** ^*^	**7.1%** ^*^	23.1%	**˂ 0,05**
Secondary middle turbinate	-	-	-	0.9%	-	0.3%	˃0,05
Maxillary sinus hypoplasia	4.5%	**5.1%** ^*^	2.8%	3.9%	**0.8%** ^*^	2%	**˂ 0,05**
Secondary superior concha	-	-	0.6%	0.6%	0.3%	0.6%	˃0,05
Ethmomaxillary sinus	**13.8%** ^*^	6.7%	**20.2%** ^*^	**4.8%** ^*^	6.8%	**9.1%** ^*^	**˂ 0,05**
Sphenomaxillary plate	**1.4%** ^*^	7.3%	**27%** ^*^	**4.5%** ^*^	8.2%	**8.2%** ^*^	**˂ 0,05**
Septum deviation	**44.4%** ^*^	**43.3%** ^*^	-	**20.9%** ^*^	**26.2** ^*^	0.8%	**˂ 0,05**
Internal carotid artery bulging	**2.3%** ^*^	**4.8%** ^*^	23.9%	**7.3%** ^*^	**7.5%** ^*^	28.2%	**˂ 0,05**
Onodi cell	18.6%	8.2%	10.4%	12.7%	14.1%	11.9%	˃0,05
Anterior clinoid pneumatization	3.4%	4.5%	4.8%	4.2%	3.1%	6.5%	˃0,05
Posterior clinoid pneumatization	2.5%	2.3%	4.5%	2.3%	1.7%	5.1%	˃0,05
Pterygoid plates pneumatization	6.7%	13.2%	32.6%	9.2%	12%	30.2%	˃0,05

**Fig. 5:**
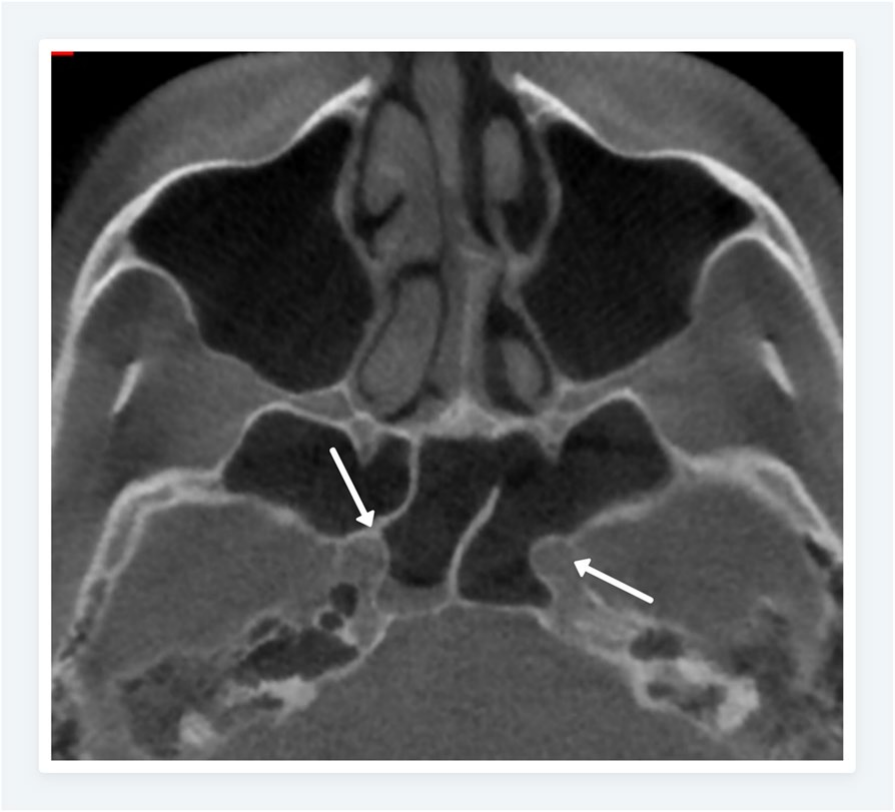
Axial CBCT scan showing bilateral internal carotid artery protrusion (arrows) into the sphenoid sinus with complete accessory septation (on the right side) inserting onto the internal carotid artery bony wall

**Fig. 6:**
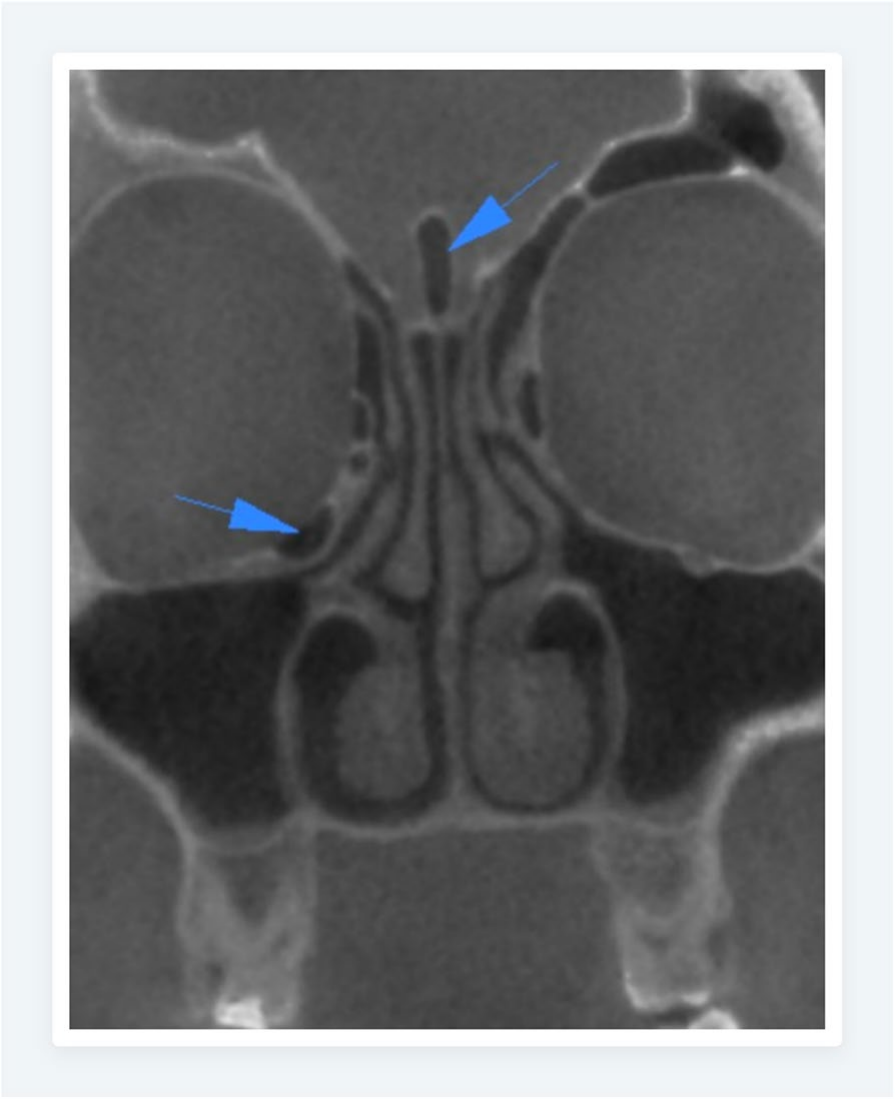
Coronal CBCT image of the Haller cell (on the right side) and crista Galli pneumatization with arrows

**Fig. 7:**
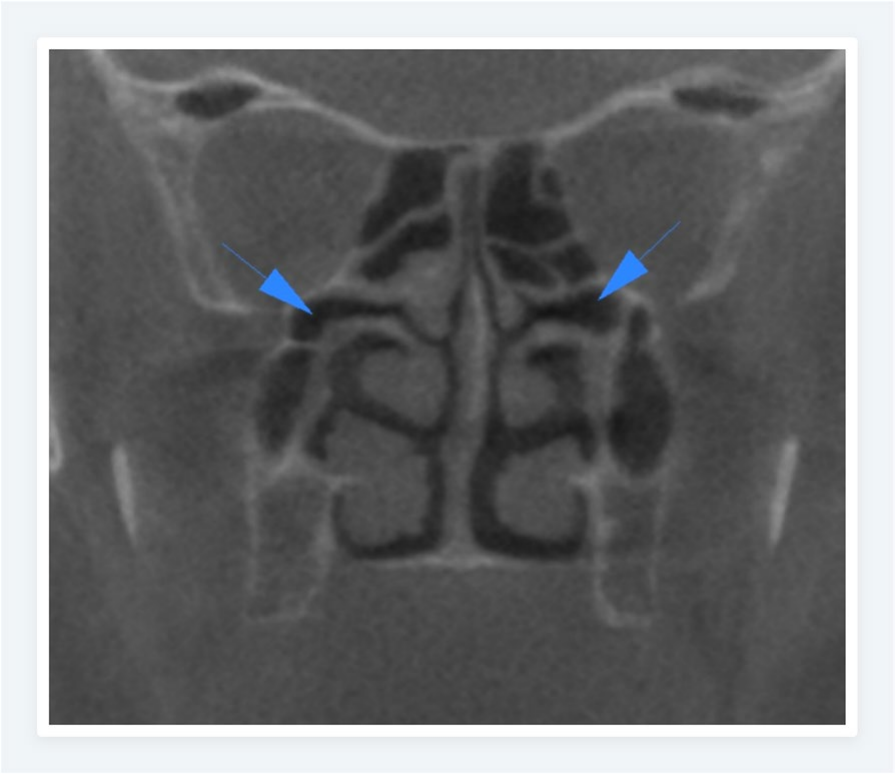
Coronal CBCT image showing the bilateral ethmomaxillary sinuses which drain into the superior meatus

Many anatomic variations were found to show substantial differences among both populations. Incidence rates of hyperpneumatization, septum pneumatization, supraorbital ethmoid cells, concha bullosa, uncinate bulla, and internal carotid artery bulging (Fig. 5) in the sphenoid sinus were significantly higher in the Turkish Cypriot group, while the incidence of Haller cell (Fig. 6), frontal sinus hypoplasia, maxillary sinus hypoplasia (Fig. 1), ethmomaxillary sinus (Fig. 7), sphenomaxillary plate, and septum deviation were significantly higher in Polish population (*p*˂0.05). No statistically significant differences were observed for other anatomic variations among the two groups (*p* > 0.05).

Minor changes occurred when the female (Table 3) and male patients (Table 4) were analyzed separately within the groups. While Haller cell and maxillary sinus hypoplasia did not significantly differ among the male patients, nasal septum pneumatization, Haller cell and internal carotid artery bulging into the sphenoid sinus did not significantly differ between the populations for female patients.

**Table 3:** Comparison of the anatomic variations in female patients

Variations	Polish /female			Turkish Cypriot/female			P value
Frontal sinus hyperpneumatization	**5.45%**			**18.37%** ^*^			**˂ 0,05**
Frontal sinus hypoplasia	**16.78%** ^*^			**9.09%** ^*^			**˂ 0,05**
Interfrontal sinus septa cell	53.85%			62.16%			˃0,05
Crista galli pneumatization	4.09%			4.71%			˃0,05
Septum pneumatization	44.8%			53.97%			˃0,05
	**Right**	**Left**	**Both**	**Right**	**Left**	**Both**	
Agger nasi cell	15.84%	12.22%	56.56%	16.56%	14.01%	52.23%	˃0,05
Supraorbital ethmoid cell	**3.18%** ^*^	**3.64%** ^*^	25.45%	**9.09%** ^*^	**11.69%** ^*^	32.47%	**˂ 0,05**
Inferior concha bullosa	1.36%	0.9%	2.26%	0.53%	1.06%	-	˃0,05
Concha bullosa	11.76%	14.48%	**32.13%** ^*^	13.23%	12.7%	**43.92%** ^*^	**˂ 0,05**
Superior concha bullosa	10.86%	9.05%	22.17%	8.7%	14.13%	16.3%	˃0,05
Haller cell	9.5%	7.24%	25.79%	7.94%	9.52%	17.46%	˃0,05
Uncinate bulla	**3.62%** ^*^	**1.81%** ^*^	**18.1%** ^*^	**13.1%** ^*^	**7.65%** ^*^	**25.14%** ^*^	**˂ 0,05**
Secondary middle turbinate	-	-	-	1.08%	-	0.54%	˃0,05
Maxillary sinus hypoplasia	4.52%	**5.43%** ^*^	1.81%	3.21%	0.53%^*^	2.14%	**˂ 0,05**
Secondary superior concha	-	-	0.45%	1.11%	0.56%	-	˃0,05
Ethmomaxillary sinus	16.29%^*^	5.88%	20.81%^*^	4.89%^*^	5.98%	9.78%^*^	**˂ 0,05**
Sphenomaxillary plate	2.26%	8.14%	30.32%^*^	4.32%	9.19%	12.97%^*^	**˂ 0,05**
Septum deviation	47.06%^*^	40.27%^*^	-	19.58%^*^	24.87%^*^	1.06%	**˂ 0,05**
Internal carotid artery bulging	2.26%	4.98%	20.81%	5.85%	6.91%	23.94%	˃0,05
Onodi cell	19.09%	8.64%	10%	13.44%	12.37%	10.22%	˃0,05
Anterior clinoid pneumatization	2.27%	5%	3.64%	5.85%	2.66%	3.72%	˃0,05
Posterior clinoid pneumatization	2.73%	2.73%	2.27%	1.6%	1.06%	3.72%	˃0,05
Pterygoid plates pneumatization	6.33%	14.48%	33.94%	7.45%	11.7%	30.32%	˃0,05

**Table 4:** Comparison of the anatomic variations in male patients

Variations	Polish /male			Turkish Cypriot/male			*P* value
Frontal sinus hyperpneumatization	2.96%^*^			28.33%^*^			**˂ 0,05**
Frontal sinus hypoplasia	16.67%^*^			5.19%^*^			**˂ 0,05**
Interfrontal sinus septa cell	40.74%			46.67%			˃0,05
Crista galli pneumatization	4.44%			3.27%			˃0,05
Septum pneumatization	42.96%^*^			54.12%^*^			**˂ 0,05**
	**Right**	**Left**	**Both**	**Right**	**Left**	**Both**	
Agger nasi cell	16.3%	11.85%	52.59%	11.9%	11.9%	56.35%	˃0,05
Supraorbital ethmoid cell	2.96%^*^	4.44%^*^	25.19%^*^	6.4%^*^	12.8%^*^	46.4%^*^	**˂ 0,05**
Inferior concha bullosa	-	0.74	-	1.18%	1.76%	-	˃0,05
Concha bullosa	9.63%^*^	8.15%^*^	31.11%	15.88%^*^	13.53%^*^	37.06%	**˂ 0,05**
Superior concha bullosa	7.41%	8.15%	15.56%	8.88%	11.83%	20.12%	˃0,05
Haller cell	6.67%	10.37%	20.74%	10.65%	13.02%	13.61%	˃0,05
Uncinate bulla	3.7%^*^	**2.22%** ^*^	24.44%	10.12%^*^	6.55%^*^	20.83%	**˂ 0,05**
Secondary middle turbinate	-	-	-	0.59%	-	-	˃0,05
Maxillary sinus hypoplasia	4.44%	4.44%	4.44%	4.73%	1.18%	1.78%	˃0,05
Secondary superior concha	-	-	0.74%	-	-	1.19%	˃0,05
Ethmomaxillary sinus	9.63%^*^	8.15%	**19.26%** ^*^	4.73%^*^	7.69%	8.28%^*^	**˂ 0,05**
Sphenomaxillary plate	-^*^	5.93%	**21.48%** ^*^	4.73%^*^	7.1%	2.96%^*^	**˂ 0,05**
Septum deviation	40%^*^	48.15%^*^	-	22.35%^*^	27.65%^*^	0.59%	**˂ 0,05**
Internal carotid artery bulging	2.22%^*^	4.44%^*^	28.89%	8.82%^*^	8.24%^*^	32.94%	**˂ 0,05**
Onodi cell	17.78%	7.41%	11.11%	11.9%	16.07%	13.69%	˃0,05
Anterior clinoid pneumatization	5.19%	3.7%	6.67%	2.38%	3.57%	9.52%	˃0,05
Posterior clinoid pneumatization	2.22%	1.48%	8.15%	2.96%	2.38%	6.55%	˃0,05
Pterygoid plates pneumatization	7.41%	11.11%	30.37%	11.18%	12.35%	30%	˃0,05

## Discussion

Profound knowledge of anatomy, surgical landmarks, and anatomic variations of the paranasal region is the key issue and prerequisite for an effective and successful functional endoscopic sinus surgery (FESS). Determine and describing the precise anatomy, anatomical variations and pathology mostly depend on the advanced imaging methods as well as the experience and skills of the practitioner [12–15]. Previous studies focused on the assessment of anatomic-radiological risk profiles which concluded as the measurements as well as detailed evaluations can help identify those patients who are at high risk for injury [10].

The results of the previous studies indicated that climatic variables such as temperature and humidity predominantly influence the mid-facial anatomy, external nasal morphology, nasal aperture, and turbinate anatomy [19, 20]. However, the influence of climatic variables on paranasal regions was not taken into consideration in these earlier studies.

According to Köppen-Geiger world climatic map, the climate of our research areas is of the Dfb type (no dry season, generally cold winter, warm and humid summer) for the Polish population and Csa type (temperature, dry and hot summer, cool and rainy winters) for Turkish Cypriot population [21].

Noback et al. [20] found a significant correlation between nasal cavity shape and climatic variables of both temperature and humidity. Variation in nasal cavity shape is correlated with a cline from cold–dry climates to hot–humid climates, with a separate temperature and vapor pressure effect. The bony nasal cavity appears mostly associated with temperature and the nasopharynx with humidity. Similarly in our study, in the Turkish Cypriot population who lives in high temperatures with humidity, the hyperpneumatization, septum pneumatization, and ethmoids variations are more frequent than in the Polish population. This may be due to climate-related variations and a higher surface-to-volume ratio in the nasal cavity.

Hubbe et al. [22] reported significant correlations between nasal cavity measurements and temperature variables and humidity measures. Our findings indicate in line with Huber’s study that both nasal cavities together with bone/mucosa interference and sphenoid sinus anatomy might be more strongly responding to climate, possibly vapor pressure.

In this study, it was found a high incidence of Haller cell, frontal sinus hypoplasia, maxillary sinus hypoplasia, ethmomaxillary sinus, and septum deviation in the Polish population. It can be interpreted as the nasal cavity shape which follows climatic trends of the increased difficulty of air-conditioning: from hot–humid to cold–dry. The shape of the paranasal anatomy might also be related to temperature and humidity which is again in line with Hubbe’s study [22].

It should be stated that based on the results of this study, that dry and cold climates would be the most difficult to condition air, thus nasal cavity shape would follow similar adaptive trends as in the Polish population such as frontal sinus hypoplasia, maxillary sinus hypoplasia, and septum deviation. This results in contracts with Hubbe’s study [22] as in their study multiple regression analysis showed that cold climates are related to higher nasal cavities with high nasal apertures and choanae. This difference can be related to that in our study which we evaluated not just the ethmoid cavity but the frontal and maxillary sinus region as well. It can be also interpreted as nasal cavity morphology might show an increase in air-wall contact with increasing difficulty of air-conditioning in physiologically more demanding environments.

Franciscus et al. [23] found in their study that a narrower superior ethmoidal breadth in supra-Saharan populations compared with sub-Saharan Bantu groups due to temperature changes. In our study, we found hypoplasia both in the Frontal and maxillary sinus which may increase the surface/volume ratio of the circulating air. The shape, however, also increases the surface/volume ratio which is an unexpected feature in climates where air-conditioning is relatively easier. [24].

Previous studies have also concentrated on the prevalence of paranasal sinus variations and their possible influence on chronic sinusitis in specific populations. There was a limited number of studies focused on the temperature and humidity effect on the paranasal sinus area in the literature. Selçuk et al. [25] studied the maxillary, frontal and sphenoid sinus volumes as well as the frontal sinus hypoplasia, nasal septum deviation, and concha bullosa in different climate and altitude conditions, and reported no significant differences which may be attributed to the small number of patients in both groups. On the contrary Asirdizer et al. [26] stated that frontal sinus widths, anteroposterior lengths, and volume were significantly higher in the cold climate group. In another study including fully intact 41 Melanesian crania, Robinson et al. [27] evaluated the paranasal sinus variations and compare their results with the literature. Agger nasi cell was the most commonly observed anatomic variant followed by the concha bullosa and Haller cell with a prevalence of 48.8%, 30%, and 29.3% respectively. Compared with the other researchers, Agger nasi cells were found to be less common in Melanesians than Caucasoids and Mongolian. On the other hand, the carotid artery bulging into the sphenoid sinus was relatively low in Melanesians while Mongoloid have a higher incidence [27].

Agger nasi cell was frequently encountered anatomic variations in both populations. Agger nasi cells are the most anterior ethmoid cells that locate anterolateral and inferior to the frontal recess. The drainage pathways of the frontal sinus are characterized by the occurrence of these cells [28, 29]. Although it is thought to be a predisposing factor for frontal sinus pathology, previous studies revealed no statistically significant correlation [4, 6, 8]. However, Sivaslı et al. [8] reported a negative correlation between the occurrence of Agger nasi cells and maxillary sinusitis and proposed these cells to be a protective barrier for maxillary sinus from descending secretion. The presence of the Agger nasi cell may also adversely affect the FESS outcomes and insufficient removal of these cells is one of the factors predicting the need for revision endoscopic sinus surgery [30, 31].

Nasal septum deviation, which normally divides the nasal cavity into nearly two equal compartments, is defined as the deviation of the bony or cartilaginous parts of the septum to right or/and left sides. The previously reported prevalence of septum deviation vary from 18 to 75.9% [4, 6, 9, 12, 17, 32]. The Polish population presented a statistically significant higher prevalence of septum deviation (87.7%) compared with the Turkish Cypriots (47.9%). Kucybała et al. [33] in 2017 reported a higher prevalence of nasal septum deviation (79.9%) in the Polish population which is very similar to our study. In another study performed by Teul et al. [34], the incidence of nasal septum deformations has been reported to be 43% in examined Polish children and adolescents. In the literature unilateral hypertrophy/extensive pneumatization of the middle turbinate has been hypothesized to play a significant role in causing contralateral nasal septal deviation [9, 32, 33]. However, studies were opposing this relationship which may be due to some of the pneumatization sizes in the middle concha being too small to cause a septum deviation, especially in the lamellar type [17, 35].

Frontal cells are an object of interest to many researchers. One of the most interesting findings of this study is that supraorbital ethmoid cells have racial differences and Turkish Cypriots have a significantly higher incidence of unilateral and bilateral supraorbital ethmoid cells. Similarly, Cho et al. [14] reported the prevalence of supraorbital ethmoid cells has racial distribution differences and its prevalence was significantly higher in the Caucasians than in the Korean population. In another study, Badia et al. [13] found ethnic differences in the prevalence of the paranasal sinus variation between the Caucasian and Chinese populations. While concha bullosa, paradoxical bending of the middle turbinate, Haller cell, and suprabullar cell were significantly higher in the London population, bent uncinated process and Onodi cell were greater in the Chinese population [13].

Many vital and critical structures surround the sphenoid sinus including the cavernous sinus, carotid arteries, optic nerve, maxillary nerve, and vidian nerve. Depending on the sphenoid sinus pneumatization type the arteries and nerves may protrude into the sinus [36]. One of the most important anatomic variations determined in this study was internal carotid artery (ICA) protrusion into the sphenoid sinus. The prevalence of the ICA protrusion varies between 8 and 41% in the previous studies [7, 12, 16, 37, 38]. The frequency of unilateral ICA protrusion in the sphenoid sinus was significantly higher in male Turkish Cypriotes, whereas there were no significant differences in female patients. Surgical risk is amplified in ICA protrusion, dehiscence of the bony canal surrounding the ICA, and presence of the septum that is inserted to the ICA prominence. Anatomic variations present significant surgical challenges and FESS or transsphenoidal approach to hypophyseal fossa might be technically difficult to perform in the presence of these variations. Intraoperative injury to ICA, hemorrhage and visual loss are the serious and less common complications of the FESS however some of them may be life-threatening.

The limitation of this study is the radiographic nature of the study that did not include any clinical symptoms and outcomes and did not evaluate the soft tissue. Although the present study was performed on Turkish Cypriote and Polish populations which are classified as Caucasian there could be genetic variations among these populations. Moreover, it would be appropriate to include the results of the similar nasal cavity and maxillary sinus variation studies conducted in different climatic regions including the Turkish and Polish populations. Further studies will be conducted to consider paranasal sinus anatomic variations in the same races with different climatic conditions.

## Conclusion

Paranasal sinus variations showed climate differences between the Turkish Cypriot and Polish populations that must be taken into account by rhinologic surgeons especially when performing frontal and sphenoid sinus surgery.

## Data Availability

The datasets used and/or analyzed during the current study are available from the corresponding author upon reasonable request.
